# Incidental PET/CT Findings of Suspected COVID-19 in a Region of High Prevalence

**DOI:** 10.7759/cureus.9716

**Published:** 2020-08-13

**Authors:** Ana M Franceschi, Michael Clifton, Osama Ahmed, Robert Matthews, Dinko Franceschi

**Affiliations:** 1 Radiology, Donald and Barbara Zucker School of Medicine at Hofstra - Northwell Health, New York, USA; 2 Radiology, Stony Brook University Hospital, Stony Brook, USA

**Keywords:** fdg, pet/ct, covid-19, sars-cov-2 infection

## Abstract

We describe a case of suspected COVID-19 pneumonia in a 61-year-old male with known primary central nervous system diffuse large B-cell lymphoma (DLBCL) who underwent restaging PET/CT during the initial peak of infection of COVID-19 pneumonia within the New York region. At the time of his routine PET-CT to assess for disease progression, typical CT imaging features of COVID-19 pneumonia were identified. Upon further investigation, the patient was asymptomatic, and his infection status remained unknown.

He was subsequently lost to follow-up with his COVID-19 status pending.

## Introduction

Coronavirus disease of 2019 (COVID-19) is a novel coronavirdae virus which causes severe acute respiratory syndrome coronavirus 2 (SARS-CoV-2). COVID-19 predominantly manifests as an atypical pneumonia features by nonproductive cough, chest pain, shortness of breath, fever and malaise. The outbreak originated in Wuhan Provence of China, and has now spread to much of the globe, infecting over 16.5 million people worldwide [1]. The United States to date have approximately 4.4 million confirmed cases and nearly 150,000 deaths.

COVID-19 causes an atypical pneumonia that can be diagnosed by chest radiograph or chest CT imaging. Both modalities illustrate multifocal bibasilar predominant patchy airspace opacities that may progress to confluent consolidations in later stages [2-3]. As these airspace opacities evolve, progressive atypical respiratory system distress ensues, with the resulting hypoxemia causing a cataclysmic cascade of multiorgan distress and eventual multiorgan failure in some patients [4-6]. This end stage severe inflammatory response has been categorized as cytokine storm syndrome (CSS). This multi-organ inflammatory process seems to be triggered by the massive intravascular release of pro-inflammatory cytokines such as interleukin-6 (IL-6) into the bloodstream, contributing to prevalence of diffuse endovascular injury, with significant microthrombi burden reported on autopsy series [7-9].

This diffuse microthrombi burden combined with endothelial destabilization results in a severe multifocal inflammatory response, noted by the histological presence of megakaryocytes, platelets, fibrin, neutrophils and other inflammatory cells [9]. As the viral particles enter the cells, primarily through the angiotensin converting enzyme ACE2 receptor [10], viral replication begins to overwhelm the cellular structure, inciting a pro-inflammatory state that disrupts the infected and adjacent endothelium. Similar histopathological findings were noted in the SARS outbreak in Southeast Asia during 2003, which was attributed to a related virus of the coronavirdae family [4]. To date, COVID-19 infection predominantly affects the respiratory system; however, various organ systems have been impacted such as the central nervous system (CNS), kidneys and heart, likely as a sequala of severe inflammation in the setting of microthrombi disease [7,11-12].

It is well known that fluorodeoxyglucose (FDG) positron emission tomography-computed tomography (PET-CT) imaging demonstrates increased uptake across a variety of pathological etiologies including infections, inflammatory processes, and neoplasms. For example, FDG PET-CT imaging has been demonstrated to be useful in localizing foci of infection and inflammation in patients with fever of unknown origin [13]. In cases of COVID-19 infection documented on whole-body PET/CT, interstitial pneumonia is the predominant imaging manifestation, with multifocal bibasilar predominant ground glass opacities demonstrating increased FDG uptake in the lung parenchyma [14-15]. The increased FDG uptake is nonspecific, however the etiology remains primarily infectious/inflammatory rather neoplastic.

## Case presentation

A 61-year-old male with a history of primary diffuse large B-cell lymphoma (DLBCL) of the CNS was treated by high dose BEAM conditioning chemotherapy followed by autologous hematopoietic stem-cell transplantation. A restaging top of head to mid-thigh FDG PET-CT was performed on March 30th, 2020. The study was performed in an outpatient imaging center within the New York region during a time of high prevalence of COVID-19 infection.

**Figure 1:**
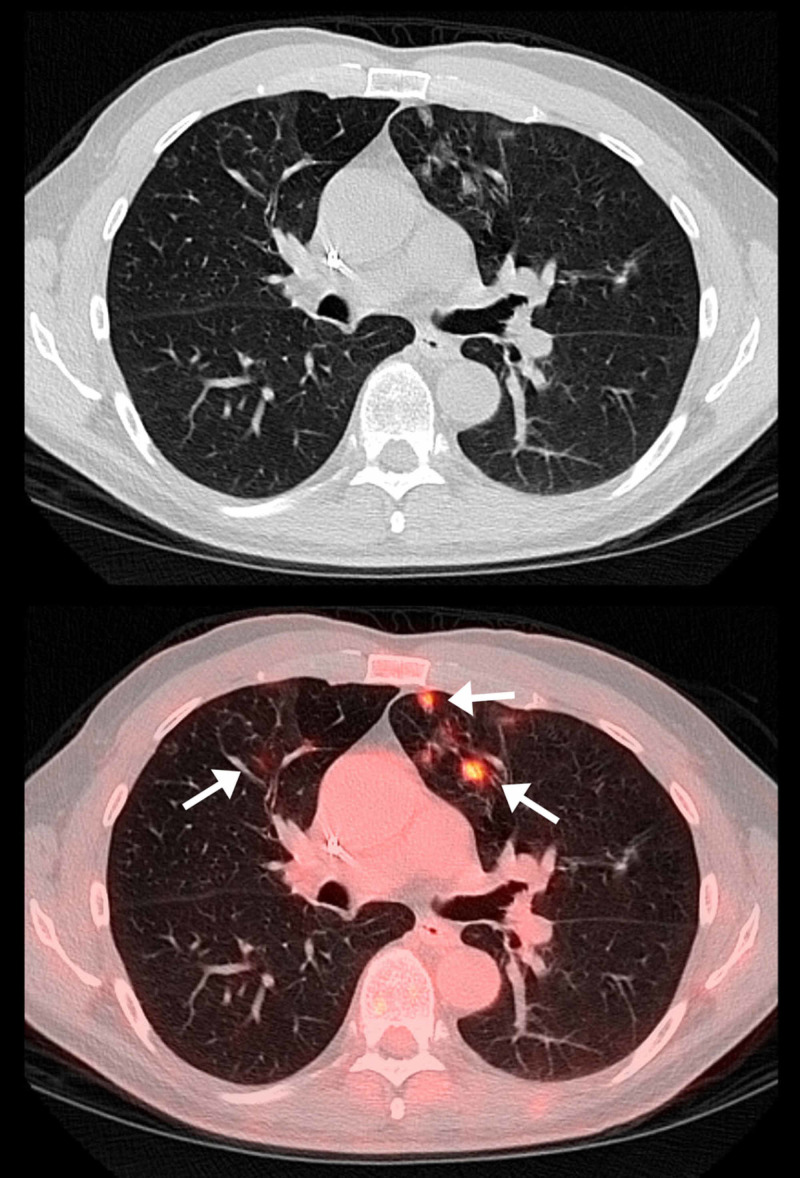
Selected axial CT and PET-CT fusion images. Selected axial CT and PET-CT fusion images demonstrate multiple, predominantly peripheral ground-glass opacities within the left upper lobe (arrow) with more subtle ground glass opacities in the right upper lobe with increased fluorodeoxyglucose (FDG) uptake (arrow).

**Figure 2:**
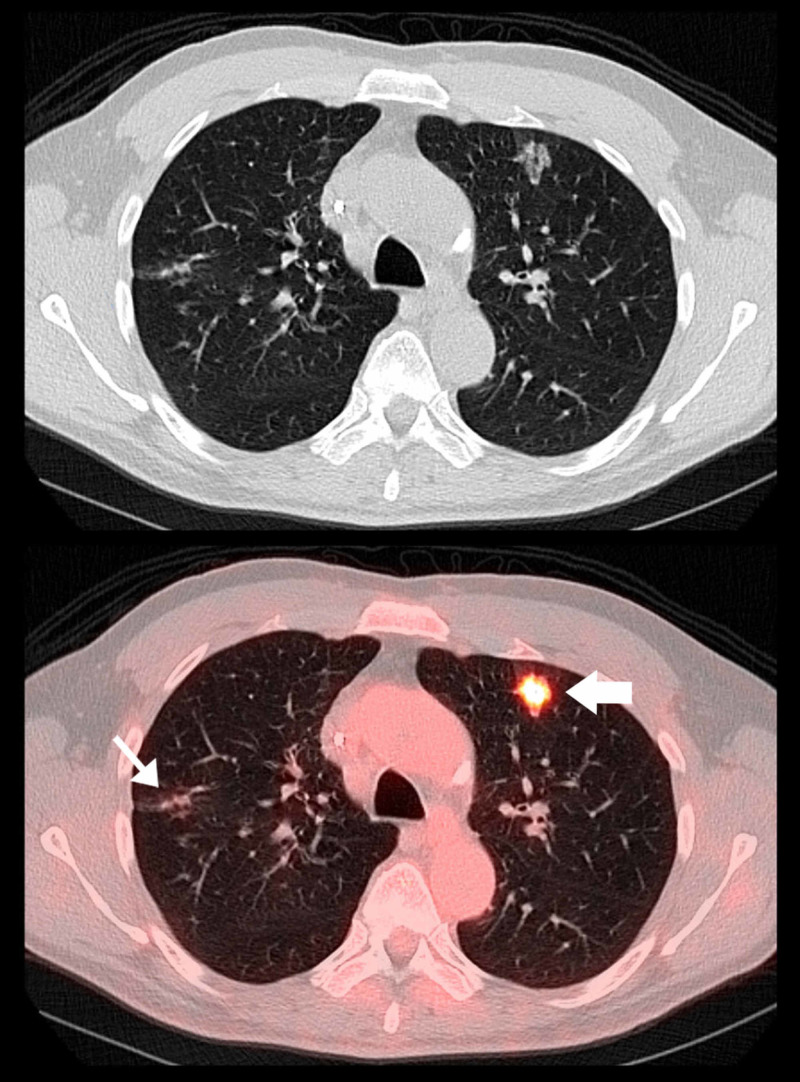
Selected axial CT and axial PET-CT fusion images. Selected axial CT and axial PET-CT fusion images through the mid chest level in lung windows demonstrate a peripheral, rounded ground-glass opacity with reticular characteristics and an air bronchogram in the left upper lung lobe (wide arrow). In the right upper lobe, a reticular ground glass opacity is also seen (thin arrow). The lesions also demonstrated increased fluorodeoxyglucose (FDG) uptake measuring up to standardized uptake value (SUV) 6.7.

**Figure 3:**
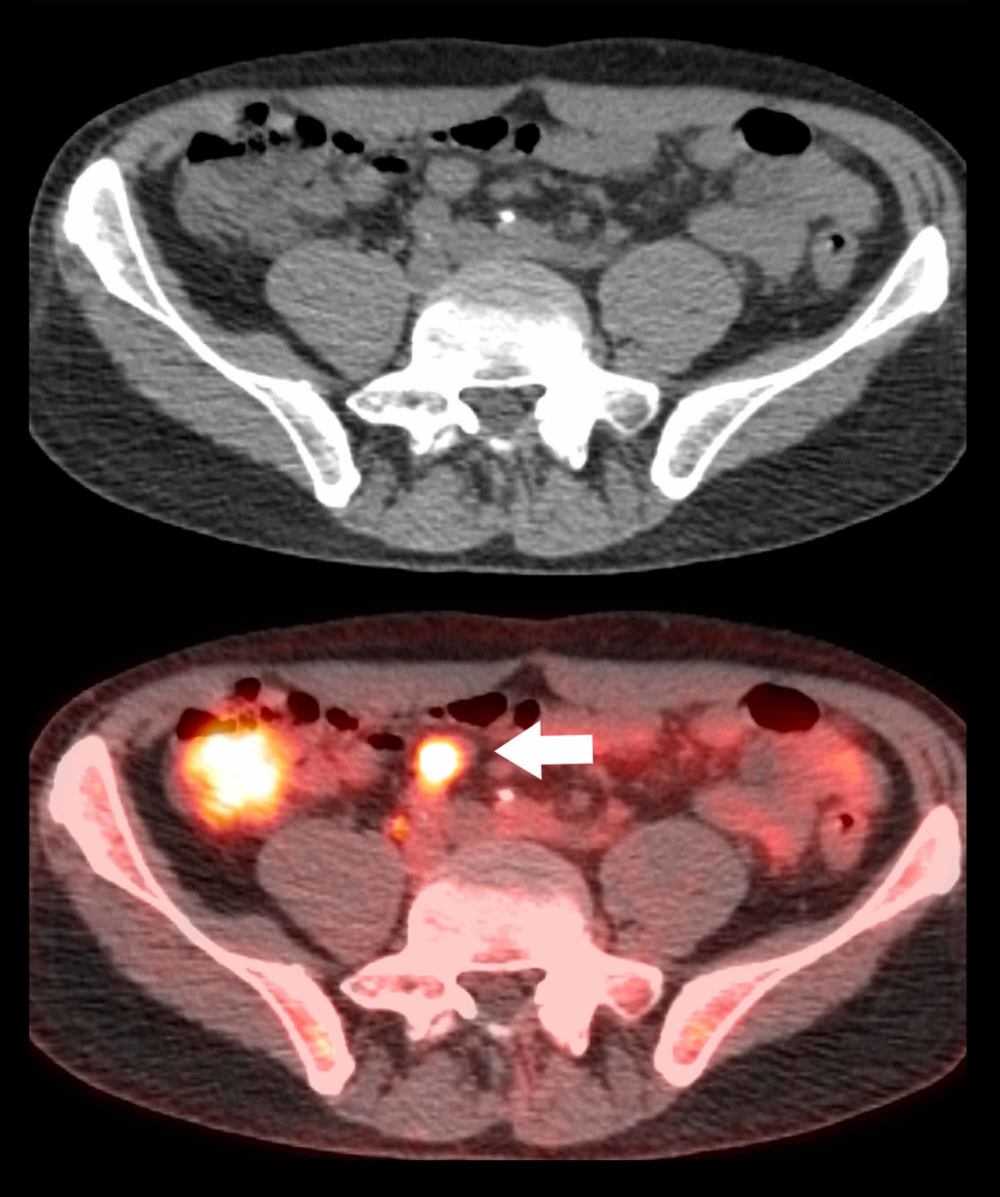
Axial CT and PET-CT fusion images. Axial CT and PET-CT fusion images show an enlarged hypermetabolic lymph node within the mesenteric region of the pelvis (wide arrow) with standardized uptake value (SUV) 6.8 which represents recurrent lymphoma.

As an incidental finding, the lung parenchyma on CT demonstrated interval development of multifocal, bibasilar and peripheral predominant, ground-glass opacities with areas of reticulation within the upper lobes demonstrating prominent FDG uptake (Figures 1-3). The patient however remained asymptomatic. Given the current high prevalence of COVID-19 within the New York region, early interstitial pneumonia related to underlying severe acute respiratory syndrome coronavirus 2 (SARS-CoV-2) infection was given a high consideration. The patient’s COVID-19 testing status remained unknown and he was subsequently lost to follow-up.

## Discussion

Typical findings of COVID-19 pneumonia include multilobar, bilateral ground glass opacities with or without consolidation [2-3]. These most commonly occur in the posterior or peripheral lungs. The ground glass opacities typically have a rounded or reverse halo morphology. Other lung findings include a reticular pattern representing thickened interstitial structures isolated or superimposed on ground glass opacities, air bronchograms, bronchiectasis, and pleural thickening. Atypical findings of COVID-19 pneumonia are isolated and/or segmental consolidations, nodules, lung cavitation and pleural effusions would suggest alternative diagnoses.

Various risk factors have been identified in predictors of morbidity and mortality for those with COVID-19 infection including advanced age, obesity, preexisting cardiovascular or cerebrovascular diseases, as well as underlying immunosuppression, including in the setting of malignancy and oncologic therapeutics [16]. Given that whole body FDG PET-CTs are largely obtained for cancer follow-up and restaging purposes, this patient population is at significantly higher risk of morbidity and mortality in the setting of SARS-CoV-2 infection.

Furthermore, according to a recent report by Albano et al., six of 65 (9%) patients undergoing restaging PET/CT for various malignancy demonstrated incidental interstitial pneumonia on CT with corresponding FDG uptake, suggesting SARS-CoV-2 infection may not be infrequent even in asymptomatic patients [14]. In addition, similar findings have been reported on PET-CT in COVID-19 patients displaying FDG-avid ground glass opacities, including some reported cases of FDG-avid mediastinal lymph nodes without significant nodal enlargement [14-15]. Moreover, the false negative rate of reverse transcriptase polymerase chain reaction (PCR)-based SARS-CoV-2 screening and testing in early asymptomatic infection, particularly in the time frame of March 2020, was a striking 38% according to a recent study by Kucirka et al. published in Annals of Internal Medicine in May 2020 [17]. Many of the typical radiological findings of early COVID-19 interstitial pneumonia are well-illustrated in our patient. Additionally, there is noteworthy increased FDG uptake within the affected parts of the lung demonstrating findings classically described in the setting SARS-CoV-2 mediated pneumonia (Figures 1, 2). Although the testing status of our patient remains unknown, it is highly suggestive that our patient was indeed infected with the novel coronavirus of 2019, especially giving his baseline immunocompromised state making him a high-risk and susceptible patient meeting CDC guidelines for an early asymptomatic person under investigation (PUI) for COVID-19 pneumonia [18].

Therefore, in regions of high prevalence of COVID-19, several considerations should be made by nuclear medicine staff for oncologic patients undergoing restaging PET/CT scans in order to minimize potential exposure to COVID-19 of this extremely vulnerable patient population [19-20]. Screening should be performed as a significant portion of the population may be asymptomatic as highlighted in the case presented here. Additionally, all equipment should be properly disinfected after scans including the computers, imaging gantry, desks and tables. Nuclear medicine staff should also have access to adequate personal protective equipments (PPEs) to protect themselves and to decrease transmission risk to vulnerable patients.

## Conclusions

We describe a case of suspected COVID-19 pneumonia in a 61-year-old male with primary DLBCL of the central nervous system who underwent routine restaging PET-CT during the initial peak of COVID-19 infection in the New York region.

The patient demonstrated classic imaging findings of early interstitial pneumonia on PET/CT, meeting radiologic diagnostic criteria of SARS-Cov-2 infection on chest imaging. Therefore, taking into consideration the ongoing high prevalence of COVID-19 in the New York region and the patient’s underlying immunocompromised state, early interstitial pneumonia related to underlying SARS-CoV-2 infection was given a high consideration. Although our patient lacked a confirmatory positive PCR result, these typical imaging features should be kept in mind when imaging oncologic patients at high risk for COVID-19 exposure.
